# Rate of venous thromboembolism in a prospective all-comers cohort with COVID-19

**DOI:** 10.1007/s11239-020-02202-8

**Published:** 2020-07-02

**Authors:** Marina Rieder, Isabella Goller, Maren Jeserich, Niklas Baldus, Luisa Pollmeier, Luisa Wirth, Alexander Supady, Christoph Bode, Hans-Jörg Busch, Bonaventura Schmid, Daniel Duerschmied, Nadine Gauchel, Achim Lother

**Affiliations:** 1 Department of Medicine III (Interdisciplinary Medical Intensive Care), Medical Center, Faculty of Medicine, University of Freiburg, Freiburg, Germany; 2 Department of Cardiology and Angiology I, Heart Center, University of Freiburg, Hugstetter Strasse 55, 79106 Freiburg, Germany; 3 Department of Emergency Medicine, Faculty of Medicine, University Hospital of Freiburg, University of Freiburg, Freiburg, Germany; 4 Institute of Experimental and Clinical Pharmacology and Toxicology, Faculty of Medicine, University of Freiburg, Freiburg, Germany

**Keywords:** COVID-19, D-dimers, SARS-CoV-2, Venous thromboembolism

## Abstract

COVID-19 is associated with a variety of clinical complications including coagulopathy, which frequently results in venous thromboembolism (VTE). Retrospective analyses reported a markedly increased rate of VTEs in COVID-19. However, most recent studies on coagulopathy in COVID-19 were only focused on critically ill patients, and without suitable control groups. We aimed to evaluate the rate of VTEs in an all-comers cohort with suspected COVID-19 during a 30-days follow-up period. We also studied the level of D-dimers and their association with the course of disease. In our prospective single-center study (DRKS00021206, 03/30/2020), we analyzed 190 patients with suspected COVID-19 admitted to the emergency department between March and April 2020. Forty-nine patients were SARS-CoV-2 positive (25.8%). The 141 SARS-CoV-2-negative patients served as control group. After completion of a 30-days follow-up, VTE was diagnosed in 3 patients of the SARS-CoV-2-positive group (6.1%, amongst these 2 ICU cases) versus 5 patients in the SARS-CoV-2-negative group (3.5%), however the difference was not statistically significant (p = 0.427). 30-days mortality was similar in both groups (6.1% vs. 5%, p = 0.720). Disease severity correlated with the maximum level of D-dimers during follow-up in COVID-19. The rate of VTE was numerically higher in SARS-CoV-2 positive all-comers presenting with suspected COVID-19 as compared to well-matched controls suffering from similar symptoms. VTEs in the COVID-19 group predominantly occurred in ICU courses. The maximum level of D-dimers during follow-up was associated with disease severity in COVID-19, whereas the level of D-dimers at admission was not.

## Highlights

- Previous studies reported an increased incidence of VTEs in COVID-19.
- These previous studies were mostly retrospective, focused on critically ill patients and did not include suitable control groups.
- In our prospective all-comers registry, the rate of VTEs was numerically higher in COVID-19 compared to a control group of patients presenting with similar symptoms, however the difference was not statistically significant.
- The maximum level of D-dimers during follow-up was associated with disease severity in COVID-19, whereas the level of D-dimers at admission was not.

## Introduction

The novel coronavirus disease 2019 (COVID-19) first emerged in Wuhan, Province Hubei, China, in December 2019 and became pandemic since then. An infection with the disease-causing virus SARS-CoV-2 can lead to a broad spectrum of clinical presentations, ranging from asymptomatic or mild cases to severe or even life-threatening courses with acute respiratory distress syndrome [1–3]. Since first case series from Wuhan reported an association between pulmonary embolism and COVID-19 [4], there is emerging evidence that besides pneumonia, coagulopathy is a common finding in patients suffering from COVID-19, especially in severe courses [5]. The occurrence of venous thromboembolism in serious COVID-19 infection is reported to be up to 60% [6–8] and the degree of coagulopathy was reported to be associated with disease severity [9]. However, the underlying pathomechanism causing this procoagulative state is not fully understood. It is suggested that hyperinflammation and hypoxemia lead to endothelial dysfunction and as a consequence to enhanced risk of thrombosis [10, 11]. Interestingly, sufficient anticoagulation in severe cases reduces the risk for venous thromboembolism and is associated with a better prognosis, whereas mild or moderate courses did not seem to profit from anticoagulation [7, 12]. In general, there is a high rate of thromboembolic events despite therapeutic anticoagulation [7], however anticoagulation is strongly recommended [13].

Common laboratory markers for venous thromboembolism are D-dimers. These are cross-linked fibrin derivatives that are formed during thrombolysis. Although they are highly sensitive for venous thromboembolism, high concentrations also occur in other disorders such as infections and inflammatory environments [14]. Several studies reported elevated D-dimers in severe COVID-19 cases and their association to worse outcomes [15–19]. However, most of these studies only retrospectively analyzed COVID-19 patients undergoing ICU-therapy [20]. There is only scant data on the rate of VTE or the prognostic and diagnostic relevance of D-dimers at hospital admission and in patients on normal wards or in non-hospitalized patients.

In this study we aimed to evaluate the rate of thromboembolic events and the prognostic relevance of D-dimers in a prospective all-comers cohort of patients with suspected COVID-19. Those patients that were finally tested as positive were compared to the SARS-CoV-2 negative patients presenting with similar symptoms.

## Patients and methods

We here report data from an investigator-initiated, single-center prospective registry study to evaluate biomarkers associated with COVID-19 (DRKS00021206, *Deutsches Register klinische Studien* (DRKS)) conducted at the University Medical Center—University of Freiburg.

The protocol of this study conforms to the ethical guidelines of the 1975 Declaration of Helsinki and was approved by the institutional ethical committee of the University of Freiburg (EK 153/20).

### Study population

All-comers admitted to the department of emergency medicine of the University Medical Center—University of Freiburg due to suspected or proven infection with SARS-CoV-2 were eligible for inclusion. The decision to perform a PCR-test for SARS-CoV-2 was made independently of study inclusion by the treating physician. Patients were asked to participate before the test results were available. Written informed consent was obtained prior to inclusion.

If patients agreed to participate, characteristics such as medical history, clinical symptoms or previous medication were recorded. Moreover, a broad spectrum of laboratory values, amongst others D-dimers, was obtained to create a biomarker profile of all study participants at the time point of hospital admission. The severity of illness was assessed in all patients using the *Sequential Organ Failure Assessment* (SOFA) score [21, 22].

Patients with a positive PCR-test for SARS-CoV-2 were finally allocated to the “positive” group, patients with a negative PCR-test for SARS-CoV-2 to the control group.

We performed a standardized 30-days follow-up period after study inclusion. All clinical data gathered during this period was obtained from the electronic patient file. During follow-up, no interventions were applied for the purpose of this study and all therapeutic and diagnostic procedures were applied as part of standard care at the discretion of the treating physicians. Finally, participants were contacted by phone and asked about the course of disease.

**Fig. 1:**
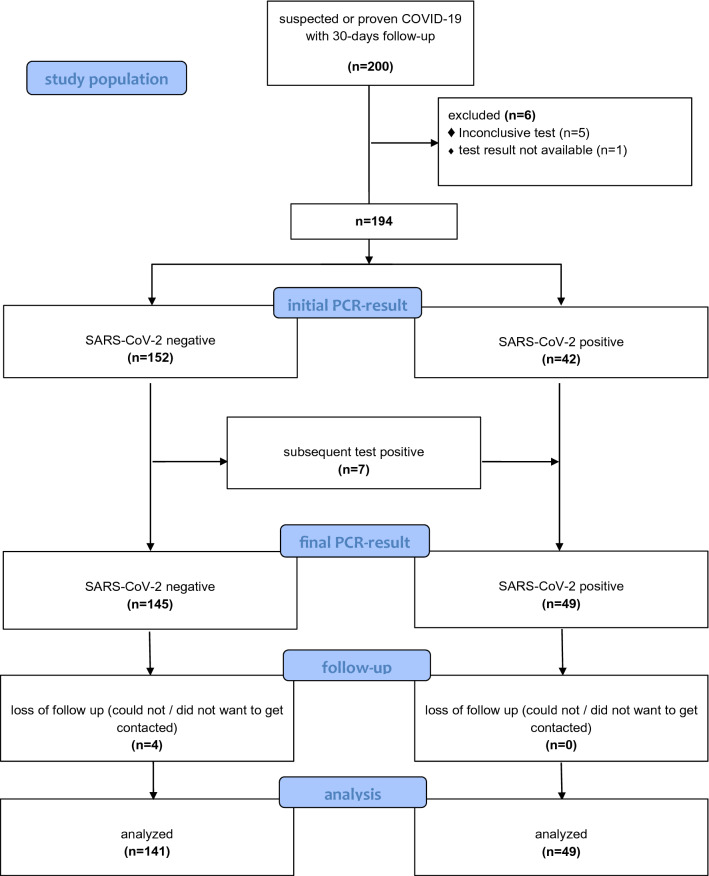
Schematic representation of the allocation to the positive or negative group of the first 200 participants included in our study. Six patients had to be excluded due to inconclusive or non-available test results. Seven of the initially negative group had a subsequent positive PCR result and had to be allocated to the positive group afterwards. Four participants of the SARS-CoV-2 negative group were lost of follow-up, while all positive patients completed follow-up. Finally, 141 negative and 49 positive participants were included in the analysis. The flow diagram is based on the template of the CONSORT flow diagram [30–32]

As requested from our ethics committee, we performed an interim analysis after the first 200 patients included in our registry had completed follow-up. These participants were recruited between the 26th of March 2020 and the 20th of April 2020. We had to exclude 6 patients because of inconclusive or non-available results of PCR-test. Of the 194 patients with a valid test, 42 were initially tested positive for SARS-CoV-2. Some of the patients from the SARS-CoV-2 negative group underwent further PCR-tests within the first days after study inclusion due to continuously suspected COVID-19. Finally, 7 of the initially negative group had a subsequent positive PCR result and had to be allocated to the positive group afterwards. In total, 49 of the 194 patients were SARS-CoV-2 positive, 145 patients were SARS-CoV-2 negative. We conducted a 30-days follow-up. 4 patients in the negative cohort were lost of follow-up as they did not want to be contacted or could not get contacted, while all positive patients completed the follow-up period. Finally, a total of 190 patients could be included in our analysis (Fig. 1).

### Endpoint

The primary outcome for this analysis was the occurrence of venous thromboembolic events (VTE). VTE included pulmonary embolism (PE); deep vein thrombosis (DVT) or venous thrombosis at other sites diagnosed by accepted imaging tests. During the period considered for the present analysis, no VTE screening strategy among COVID-19 patients was in place at the study site: VTE imaging tests were only performed at the discretion of the treating physician in subjects with signs or symptoms of DVT or with an unexplained clinical worsening of e.g. the respiratory function or a rapid increase of D-dimer levels.

The length of in-hospital-/ICU-stay as well as the days on ventilation and their correlation to the maximum D-dimer levels were considered as secondary outcome measures.

### Data analysis

For analysis, data were blinded to patient identity. Statistical analyses were performed using SPSS (version 25, IBM, SPSS Statistics, Armonk, USA) and GraphPad Prism 5 (GraphPad Software, San Diego, USA). Statistically significant outliers were excluded using Gubb’s test. Continuous variables were tested for normal distribution by using the Shapiro–Wilk test. Data are presented as mean ± standard deviation if found to follow a Gaussian distribution or otherwise as median with interquartile range.

Variables following Gaussian distribution were compared using student’s t-test, non-normally distributed continuous values by using Mann–Whitney-U test. Categorical variables were assessed by chi-square test or Fisher’s exact test as appropriate.

Correlation analysis was performed using the Spearman test for non-parametric data.

A two-tailed p-value less than 0.05 was considered statistically significant.

## Results

Of the 190 patients that were finally analyzed, 49 were SARS-CoV-2 positive and 141 negative. The median age in both groups was 60 years. 61.2% of the SARS-CoV-2 positive patients were men compared to 50.4% in the SARS-CoV-2 negative group; yet the difference was not statistically significant. The SOFA-Score did not differ between both groups, indicating similar grades of illness.

We considered body mass index (BMI), smoking status, known oncological disease and previous VTE as risk factors for the development of VTEs within the study period and analyzed these parameters. There was no significant difference in any of these parameters, indicating a comparable thrombotic risk cluster in both groups. Moreover, there was no statistically significant difference in preexisting anticoagulation.

The patients differed significantly in the symptoms presented at admission: dyspnea was more common in the SARS-CoV2 negative group (41.7% vs. 52.4%, p = 0.002), whereas cough (58.3% vs. 37.6%, p < 0.0001) and fever (75% vs. 48.2%, p < 0.0001) could be observed more frequently in the COVID-19 patients. The rate of hospital admissions tended to be higher in the SARS-CoV-2 positive group (81.6% vs. 66.7%, p = 0.068) (Table 1).

**Table 1:** Patient characteristics at hospital admission

	SARS-CoV-2 positive (n = 49)	SARS-CoV-2 negative (n = 141)	p-value
Patients characteristics			
Age [years]	60 ± 23^a^	60 ± 33^a^	0.6499^c^
Sex [male]	30 (61.2%)^b^	71 (50.4%)^b^	0.245^d^
BMI [kg/m^2^]	26.6 ± 6.6^a^	24.6 ± 7,62^a^	0.3558^c^
Smoking	5 (10.6%)^b^	27 (19.4%)^b^	0.188^d^
Symptoms at admission			
Dyspnoe	20 (41.7%)^b^	74 (52.4%)^b^	0.002^d^
Cough	28 (58.3%)^b^	53 (37.6%)^b^	< 0.0001^d^
Fever	36 (75%)^b^	68 (48.2%)^b^	< 0.0001^d^
SOFA-Score	1 ± 2.75^a^	1 ± 3^a^	0.8767^c^
Medical history			
Oncological disease	11 (22.4%)^b^	46 (32.6%)^b^	0.208^d^
Previous VTE	6 (12.2%)^b^	15 (10.8%)^b^	0.887^d^
Preexisting anticoagulation	6 (12.2%)	26 (18.8%)	0.222^d^
Heparine (therapeutic)	1 (2.0%)^b^	2 (1.4%)^b^	0.594^d^
DOAK	4 (8.1%)^b^	17 (12.3%)^b^	0.592^d^
VKA	1 (2.0%)^b^	7 (5.1%)^b^	0.624^d^
Diagnostics/diagnosis at admission			
D-dimers [mg/l]	1.1 ± 1.4^a^	0.8 ± 1.7^a^	0.2995^c^
Native CT-scan	4 (8.2%)^b^	8 (5.7%)^b^	0.374^d^
CTPA	5 (10.2%)^b^	14 (9.9%)^b^	1.0^d^
VTE diagnosed at admission	0 (0%)^b^	3 (2.1%)^b^	0.570^d^
Hospital admission	40 (81.6%)^b^	94 (66.7%)^b^	0.068^d^

p-values refer to the comparison between the SARS-CoV-2 negative and the SARS-CoV-2 positive patients

^a^Presented as median ± interquartile range

^b^Number of patients (with percentage based on the number of patients with a non-missing value for that characteristic)

^c^Based on Mann–Whitney-U test for nonparametric variables

^d^Based on chi-square test/Fisher’s exact test as appropriate for categorical variables

A native CT-scan was performed in 4 patients (8.2%) in the SARS-CoV-2 positive group compared to 8 patients (5.7%) in the negative group (p = 0.374). CT-pulmonary Angiography (CTPA) was performed in 5 patients (10.2%) in the SARS-CoV-2 positive group compared to 14 patients (9.9%) in the negative group (p = 1.0). The rate of VTEs diagnosed at admission was 0 in the SARS-CoV-2 positive group vs. 3 in the SARS-CoV-2 negative group; however the difference was not statistically significant.

D-Dimers at admission did not differ between both groups (1.1 ± 1.4 mg/l vs. 0.8 ± 1.7 mg/l, p = 0.2995) (Table 1).

After completion of the 30-days follow-up period, a total of 3 VTEs had occurred in the SARS-CoV-2 positive group (6.1%). 2 of these 3 patients had suffered pulmonary embolism, 1 was diagnosed with a thrombosis of the portal vein. The two patients with the diagnosis of pulmonary embolism had been admitted to ICU (patient 1: 20 days in hospital, 9 days on ICU; patient 2: hospitalized during the whole follow-up period, 27 days on ICU). The patient with the thrombosis of the portal vein stayed in hospital for a total of 7 days, but was not admitted to ICU. In the SARS-CoV-2 negative group, a total of 5 patients were diagnosed with pulmonary embolism (3.5%). All of them had been hospitalized for at least 10 days, but none was admitted to ICU. The difference in total VTE between both groups was not statistically significant (p = 0.427). Moreover, the maximum level of D-dimers during follow-up did not differ between both groups (1.2 ± 3.3 vs. 2.1 ± 3.5, p = 0.8819).

We observed that SARS-CoV-2 positive patients stayed markedly longer in hospital (10 ± 12 vs. 5 ± 12, p = 0.0006), tended to be more often admitted to ICU (16.3% vs. 7.1%, p = 0.085) and stayed longer on ICU (16.8 ± 9.7 vs. 3.8 ± 2.4, p = 0.0038) than participants of the control group. COVID-19 patients were more often in need of invasive ventilation (12.2% vs. 2.1%, p = 0.01). The 30-days mortality however did not differ between both groups (6.1% vs. 5%, p = 0.720) (Table 2).

**Table 2:** Outcome and patient characteristics after 30 days follow-up

	SARS-CoV-2 positive	SARS-CoV-2 negative	p-value
Hospital stay			
Days at hospital	10 ± 12^a^	5 ± 12^a^	0.0006^e^
Admitted to ICU	8 (16.3%)^c^	10 (7.1%)^c^	0.085^f^
Days on ICU	16.8 ± 9.7^b^	3.8 ± 2.4^b^	0.0038^d^
Non-invasive ventilation	3 (6.1%)^c^	2 (1.4%)	0.109^f^
Invasive ventilation	6 (12.2%)^c^	3 (2.1%)^c^	0.01^f^
VTE diagnosis/diagnostics			
Maximum level of D-dimers [mg/l] during follow-up	1.2 ± 3.3^a^	2.1 ± 3.5^a^	0.8819^e^
Native CT-scan	7 (14.3%)^c^	9 (6.4%)^c^	0.131^f^
CTPA	8 (16.3%)^c^	27 (19.1%)^c^	0.831^f^
VTE total	3 (6.1%)^c^	5 (3.5%)^c^	0.427^f^
30-days mortality	3 (6.1%)^b^	7 (5%)^b^	0.720^f^

p-values refer to the comparison between the SARS-CoV-2 negative and the SARS-CoV-2 positive patients

^a^Presented as median ± interquartile range

^b^Presented as mean ± standard deviation

^c^Number of patients (with percentage based on the number of patients with a non-missing value for that characteristic)

^d^Based on student’s t-test for variables following a Gaussian distribution

^e^Based on Mann–Whitney-U test for nonparametric variables

^f^Based on chi-square test/Fisher’s exact test as appropriate for categorical variables

As mentioned above, the levels of D-dimers at admission and the maximum level during follow-up did not differ between both groups. The D-dimers at admission did not correlate with disease severity measured as days at hospital, days on ICU and length of on non-invasive or invasive ventilation in the SARS-CoV-2 positive patients. However, the length of hospital stay positively correlated with the level of D-dimers at hospital admission in the SARS-CoV-2 negative group.

In contrast to the level of D-dimers at admission, the maximum levels of D-dimers during the 30-days follow-up period correlated significantly with the severity of disease in the SARS-CoV-2 positive group whereas they did not correlate with days at hospital, days on ICU and days on non-invasive or invasive ventilation in the control group (Table 3).

**Table 3:** Correlation analysis

	SARS-CoV-2 positive	SARS-CoV-2 negative
D-Dimers at admission		
Days at hospital	r = 0.201	**r = 0.631**
	p = 0.287	**p = < 0.001**
Days on ICU	r =− 0.32	r = 0.131
	p = 0.867	p = 0.207
Days on non-invasive ventilation	r = 0.124	r = 0.134
	p = 0.514	p = 0.197
Days on invasive ventilation	r =− 0.093	r = 0.048
	p = 0.626	p = 0.646
Maximum of D-dimers during follow-up		
Days at hospital	**r = 0.398**	r = 0.419
	**p = 0.029**	p = 0.106
Days on ICU	**r = 0.550**	r = 0.064
	**p = 0.002**	p = 0.814
Days on non-invasive ventilation	r = 0.316	r = − 0.140
	p = 0.089	p = 0.605
Days on invasive ventilation	**r = 0.439**	r = − 0.140
	**p = 0.015**	p = 0.605

The level of D-dimers at hospital admission and the maximum level during follow-up were correlated with days at hospital, days on ICU, days on non-invasive ventilation or days on invasive ventilation. As all parameters were non-normally distributed, Spearman correlation analysis was performed for all tests. A p-value less than 0.05 was considered statistically significant (bold).

## Discussion

We here report data from the first 200 all-comers with suspected or proven SARS-CoV-2 infection included in our prospective single-center registry study to evaluate biomarkers associated with COVID-19. We analyzed the data in regards of VTEs and evaluated the association between D-dimers and disease severity.

Our study collective consisted of all-comers presenting at the emergency department with symptoms such as fever, cough or dyspnea that were suspicious of COVID-19. As most currently available data on COVID-19 is based on retrospective analyses, the prospective design and the comparison between SARS-CoV-2 positive patients to a control collective presenting with similar symptoms are a strength of our study.

All patients analyzed for this manuscript were included in the study during the peak of the COVID-19 pandemic in Germany in March/April 2020. In total, ¼ of all patients presenting with the above-mentioned symptoms at our emergency department during these months suffered from COVID-19. Interestingly, more than 10% of all positive participants were initially tested negative, but received another SARS-CoV-2 test within the next days due to persistent symptoms suspicious for COVID-19 and were finally proven positive. These findings show that repeated testing is indicated if PCR result is initially negative but patients present with clinically suspected COVID-19.

In our collective, the SARS-CoV-2 positive and negative patients did not differ in most baseline characteristics. Age, sex, severity of disease assessed by SOFA-Score and risk factors for VTEs such as BMI, smoking status, preexisting anticoagulation or oncological diseases were similar in both groups. Finally, the rate of VTEs in the COVID-19 cohort was numerically higher in the SARS-CoV-2 positive group, however the difference was not statistically significant. Previous reports described that patients suffering from COVID-19 present a high rate of venous and arterial thromboembolic events, partly even despite prophylactic or therapeutic anticoagulation [7, 8, 23]. However, all these previous studies were retrospectively analyzing critically ill patients with COVID-19 requiring ICU-therapy. None of these reports was focused on all-comers and none of them had prospectively included a control collective. Yet, consistent with these previous descriptions that critically ill patients with COVID-19 frequently develop VTEs, the 2 patients of our positive group diagnosed with pulmonary embolism had been admitted to ICU prior to VTE diagnosis. None of the VTE-patients of our control group required ICU-therapy.

D-dimers at admission and the maximum levels during follow-up did not differ between our study groups. Median D-dimers at admission were 1.1 mg/dl in our COVID-19 group. This is in line with previous studies from Wuhan reporting D-dimer levels at admission between 0.2 – 1.4 mg/dl in SARS-CoV-2 positive patients [24, 25].

A previous study reported elevated D-dimers in COVID-19 positive patients compared to controls [26], however the authors described COVID-19 patients in contrast to healthy controls, whereas we compared SARS-CoV-2 positive patients with negative participants presenting with similar symptoms. Moreover, the level of D-dimers in SARS-CoV-2 positive patients reported by the authors (10.36 mg/l) was markedly elevated compared to the D-dimers in our all-comers cohort that was comparable to other analyses from China as reported above. In our SARS-CoV-2 positive collective, D-dimers at admission did not correlate with disease severity depicted by days in hospital, days on ICU and days on ventilation. Previous retrospective studies from Wuhan claimed that elevated D-dimers at admission could effectively predict ICU-admission or in hospital-mortality [24, 25]. Yet these were retrospective trials with most probably selection bias, as not all patients received D-dimer testing at admission and only those that did for different reasons were included [15]. In contrast to this study, our trial had a prospective design with a control group and we determined D-dimers at admission independently of clinically suspected VTE.

The maximum level of D-dimers correlated significantly with the days in hospital, the length of ICU-stay and duration of ventilation in the COVID-19 cohort. This is in line with a variety of previous studies reporting that D-dimers were elevated in critically ill hospitalized SARS-CoV-2 patients if compared to less severe courses [18, 19, 27]. A recent pooled analysis also reported an association of D-dimer levels to disease severity [20].

The mortality in the Covid-19 group did not differ from the SARS-CoV-2 negative participants. This is in contrast to a previous retrospective analysis comparing the mortality of patients with COVID-19 pneumonia to SARS-CoV-2 negative patients with pneumonia. This retrospective analysis from Wuhan reported a markedly elevated mortality in SARS-CoV-2 positive patients compared to the controls [28]. However, the study from Wuhan was a retrospective analysis, whereas we prospectively compared COVID-19 patients to negative participants presenting with similar symptoms.

The case-fatality rate in Germany is currently 4.7% [29]. Mortality in our SARS-CoV-2 positive study group was slightly higher (6.1%), 80% of our patients were hospitalized. Compared to other parts of the world, the mortality rate in Germany is relatively lower. For example, case fatality rate in Canada is currently 8.2% and in France 15.2% [29]. A number of reasons may explain these differences, including different healthcare systems.

## Conclusion

In our prospective single-center registry study, the rate of VTEs was numerically higher in SARS-CoV-2 positive all-comers presenting with suspected COVID-19 as compared to well-matched controls suffering from similar symptoms. However, the difference in our study was not statistically significant. VTEs in the SARS-CoV-2 positive group mostly occurred in the severe courses. The maximum level of D-dimers during follow-up was associated with disease severity in COVID-19, whereas the level of D-dimers at admission was not.

### Limitations

Regarding the results of our analysis, we have to consider the comparatively low number of SARS-CoV-2 positive patients (49 in total) and the low number of participants with severe course of disease. Moreover, we did not screen our collective systematically for VTEs. Only clinically apparent VTEs were considered. However, the prospective study design comparing COVID-19 patients with a control group presenting with similar symptoms is an extraordinary strength of our study. To our knowledge, nearly all previous reports on COVID-19 were based on retrospective analyses and mostly without any control groups.

## Data Availability

All datasets used in this manuscript are available from the corresponding author on reasonable request.
